# Immunogenicity of recurrent mutations in MYD88 and EZH2 in non-Hodgkin lymphomas

**DOI:** 10.1186/2051-1426-3-S2-P386

**Published:** 2015-11-04

**Authors:** Julie S Nielsen, Andrew R Chang, Darin A Wick, Colin G Sedgwick, Zusheng Zong, Andrew J Mungall, Bemuluyigza Baraki, Natalie Kinloch, Zabrina L Brumme, Steven P Treon, Joseph M Connors, Randy D Gascoyne, John R Webb, Brian R Berry, Ryan D Morin, Nicol Macpherson, Brad H Nelson

**Affiliations:** 1 BC Cancer Agency, Victoria, BC, Canada; 2 Simon Fraser University, Burnaby, BC, Canada; 3 BC Cancer Agency, Vancouver, BC, Canada; 4 Dana-Farber Cancer Institute, Boston, MA, USA; 5 University of British Columbia, Vancouver, BC, Canada

## 

A fundamental challenge in cancer genomics is to design effective, personalized treatments based on the mutational profiles of tumors. Pharmacological targeting of the numerous aberrant pathways found in individual tumors remains exceedingly challenging, but T cell-based therapies are an attractive alternative because of the enormous diversity and exquisite specificity of antigen recognition. We assessed the immunogenicity of three common driver mutations in human lymphoma – MYD88^L265P^, EZH2^Y641N^, and EZH2^Y641F^ – to evaluate their suitability as targets for immunotherapy. Antigen presenting cells were loaded with overlapping peptide libraries containing each mutation and used to stimulate autologous T cells from healthy donors and lymphoma patients. Stimulated T cells were screened by interferon-gamma ELISPOT for reactivity to mutant versus wildtype peptides as well as full-length proteins. All three peptide libraries elicited T cell responses from multiple donors representing diverse HLA haplotypes. Moreover, we identified peptides from MYD88^L265P^ and EZH2^Y641N^ that were naturally processed and presented, and the corresponding T cell responses were specific for mutant proteins. Thus, MYD88^L265P^ and EZH2^Y641N^ both represent compelling antigens for immunotherapy of lymphoma patients. Funding was provided by the BC Cancer Foundation, the Canadian Cancer Society, and the Waldenstrom's Macroglobulinemia Foundation of Canada.

